# Audit of Internal Medicine Liaison, Documentation, and Follow-Up for Medical Comorbidities in Psychiatric Inpatients

**DOI:** 10.1192/bjo.2026.11843

**Published:** 2026-06-30

**Authors:** Muhammad Mansoor Ali, Syeda Areeba Siraj, Maryam Arshad, Ali Abbas Zahid, Muhammad Zubair

**Affiliations:** 1 Al Aleem Medical College/ Gulab Devi Hospital, Lahore, Pakistan; 2 None, Lahore, Pakistan

## Abstract

**Aims:**

To evaluate the quality and timeliness of Internal Medicine (IM) referrals, documentation, and follow-up for psychiatric inpatients with medical comorbidities; to identify gaps in liaison between Psychiatry and Internal Medicine; to implement targeted interventions; and to assess improvement following these interventions.

**Methods:**

A closed-loop clinical audit was conducted over a 4 month period in the Department of Psychiatry, Gulab Devi Teaching Hospital, Lahore. Two audit cycles were completed. A total of 35 psychiatric inpatients with identified medical comorbidities were included in each phase (pre- and post-intervention). Data were collected retrospectively from admission clerking notes, inpatient progress notes, and IM referral records.

Audit standards were based on good clinical practice for the management of medical comorbidities in psychiatric inpatients. Parameters assessed included: identification of medical comorbidities, IM referral made, documentation of indication for referral, attachment of relevant vital signs or laboratory investigations, timeliness of referral, documentation of IM review, documentation of IM management plans, and implementation of IM recommendations.

Interventions included introduction of a standardized IM referral proforma, targeted reminders to psychiatry junior doctors regarding referral and documentation, reinforcement of combined verbal and written handover of IM advice, and use of a ward-round checklist toensure review and implementation of IM recommendations. Post-intervention data were collected using the same methodology.

**Results:**

Pre-intervention (n=35):

IM referrals were made in 65.7% of eligible patients. Documentation of referral indication was present in 78.3%, and relevant investigations were attached in 60.9%. Same-day referral occurred in 39.1%. IM review was documented in 42.9%, IM management plans in 37.1%, and IM recommendations were implemented in 31.4%. Complete referral-review-action loops were achieved in 28.6% of cases.

Post-intervention (n=35):

IM referral rates increased to 88.6% (+22.9%). Documentation of referral indication improved to 93.5% (+15.2%), and attachment of relevant investigations to 87.1% (+26.2%). Same-day referrals increased to 71.0% (+31.9%). Documentation of IM review improved to 80.0% (+37.1%), documentation of management plans to 74.3% (+37.2%), and implementation of IM recommendations to 71.4% (+40.0%). Complete referral-review-action loops increased to 68.6% (+40.0%).

**Conclusion:**

This audit identified significant baseline gaps in liaison, documentation, and follow-up of medical comorbidities in psychiatric inpatients. Following simple, structured interventions implemented over a 3–4 month period, substantial improvements were observed across all domains. These findings highlight the effectiveness of low-cost, system-based measures in strengthening Psychiatry–Internal Medicine collaboration and improving patient safety. Continued re-audit is recommended to sustain improvements.

